# Comparison of PNI and GNRI nomogram models for predicting postoperative complications in elderly patients with lung cancer: a retrospective study

**DOI:** 10.3389/fnut.2026.1848923

**Published:** 2026-06-04

**Authors:** He Zemin, Zan Ziliang, Wei Qiang, Liu Keting

**Affiliations:** 1 Department of Thoracic Surgery, The First People's Hospital of Shuangliu District, Chengdu, Sichuan, China; 2 Department of Neurology, Chengdu Seventh People's Hospital, Chengdu, Sichuan, China

**Keywords:** elderly patients, GNRI, non-small cell lung cancer, PNI, postoperative complications

## Abstract

**Objective:**

This study aims to clarify the correlation between the nutritional indicators PNI and GNRI and postoperative cardiopulmonary complications in elderly patients with non-small cell lung cancer (NSCLC). The study also aims to construct an early postoperative visual prediction tool to optimize the efficacy of assessing postoperative cardiopulmonary complication risk in elderly NSCLC patients.

**Methods:**

This retrospective study analyzed the clinical data of elderly lung cancer surgery patients in our department. 364 Patients who met the inclusion criteria were assigned at a 7:3 ratio. Logistic regression analysis was performed to create a nomogram that predicts postoperative cardiopulmonary complications and identifies independent risk factors. We evaluated the model's performance using the C-index, the area under the curve (AUC), the calibration curve, and the decision curve analysis (DCA). We verified the model's stability using the validation set.

**Results:**

This study included 364 elderly patients undergoing lung cancer surgery, with a postoperative cardiopulmonary complication rate of 26.65%.Multivariate logistic regression identified smoking history, postoperative PNI, ΔPNI, preoperative GNRI, and operative duration as independent risk factors (*P* < 0.05). A nomogram was constructed based on these factors, achieving AUC values of 0.82 (95% CI: 0.76–0.88) in the training set and 0.87 (95% CI: 0.79–0.94) in the validation set. Calibration was satisfactory (H-L test, *P* > 0.05), and decision curve analysis confirmed clinical net benefit, with prediction accuracies of 76.0% and 80.0%, respectively.

**Conclusion:**

This study confirms that smoking history, nutritional indicators (postoperative PNI and ΔPNI and preoperative GNRI), and operation time are all independent risk factors for postoperative cardiopulmonary complications in elderly patients with non-small cell lung cancer (NSCLC). The predictive model based on these factors exhibits good discrimination and calibration and provides significant clinical net benefit. This model can effectively assist in the early identification of risk and the individualized perioperative intervention for postoperative cardiopulmonary complications in these patients.

## Introduction

Lung cancer is the leading cause of cancer-related deaths worldwide (1). Non-small cell lung cancer (NSCLC) accounts for the vast majority of these cases (2). As the population ages, the proportion of elderly patients with lung cancer is increasing significantly (3). However, elderly patients often face significantly higher perioperative risks than younger patients due to decreased physiological reserve, comorbidities, and impaired immune and nutritional status (4). These factors pose severe challenges to surgical decision-making. Cardiovascular and pulmonary complications are key negative events that affect the surgical outcomes of elderly patients with lung cancer (5). Studies have shown that the incidence of postoperative pulmonary complications in elderly patients can be as high as 31.1%, with pneumonia, atelectasis, and persistent air leaks being the most common (6). A retrospective analysis of elderly patients found that those who developed postoperative pulmonary complications had significantly worse 5-year overall and recurrence-free survival rates compared to those without complications (7). Another big data analysis based on common cancers in Korea indicated that the cumulative incidence of pneumonia after lung cancer surgery was the highest among all types of cancers, reaching 4.5% (8). Precise intervention hinges on identifying high-risk factors for postoperative complications. In addition to known factors such as age, chronic obstructive pulmonary disease, and the scope and duration of surgery, (9, 10) the preoperative nutritional and immune status of elderly cancer patients has received increasing attention. Malnutrition is prevalent in this population. Currently, various composite indicators that combine hematology and anthropometry are used clinically to quantitatively assess nutritional and inflammatory status. The Prognostic Nutrition Index (PNI) and the Geriatric Nutrition Risk Index (GNRI) are preferred due to the simplicity of their calculations and the accessibility of their parameters. The PNI uses serum albumin and lymphocyte count to reflect nutritional and immune levels comprehensively, while the GNRI uses serum albumin and body mass index, making it more suitable for screening nutritional risk in elderly populations. The prognostic value of both indices in predicting tumor surgery outcomes has been widely confirmed. In gastric, colorectal, and esophageal cancers, as well as other tumors, a low preoperative PNI or GNRI is associated with a higher incidence of postoperative complications and worse survival outcomes (11–14).

Multicenter international studies in the field of lung cancer have shown that a low preoperative PNI is an independent predictor of increased postoperative complications and can affect long-term survival and the response to postoperative drug treatment (15).

While the value of the preoperative nutritional index (PNI) and global nutritional risk index (GNRI) in predicting long-term survival in cancer patients is widely accepted, their association with specific postoperative and short-term cardiopulmonary complications in elderly patients with non-small cell lung cancer (NSCLC) is not fully understood (16). Existing studies often analyze complications as a single overall endpoint, lacking targeted exploration of cardiopulmonary complications, which are key outcomes directly affecting early recovery. In clinical practice, assessing postoperative risks for elderly lung cancer patients still relies heavily on empirical judgment or single clinical factors. There are no specialized, visualized prediction tools that integrate multidimensional information, such as nutritional status. Thus, this study aims to explore the relationship between preoperative PNI and GNRI and postoperative cardiopulmonary complications. The study also aims to construct and validate an individualized prediction model and provide scientific evidence for perioperative risk assessment and precision intervention in elderly lung cancer patients.

## Methods

### Patients

**Figure 1:**
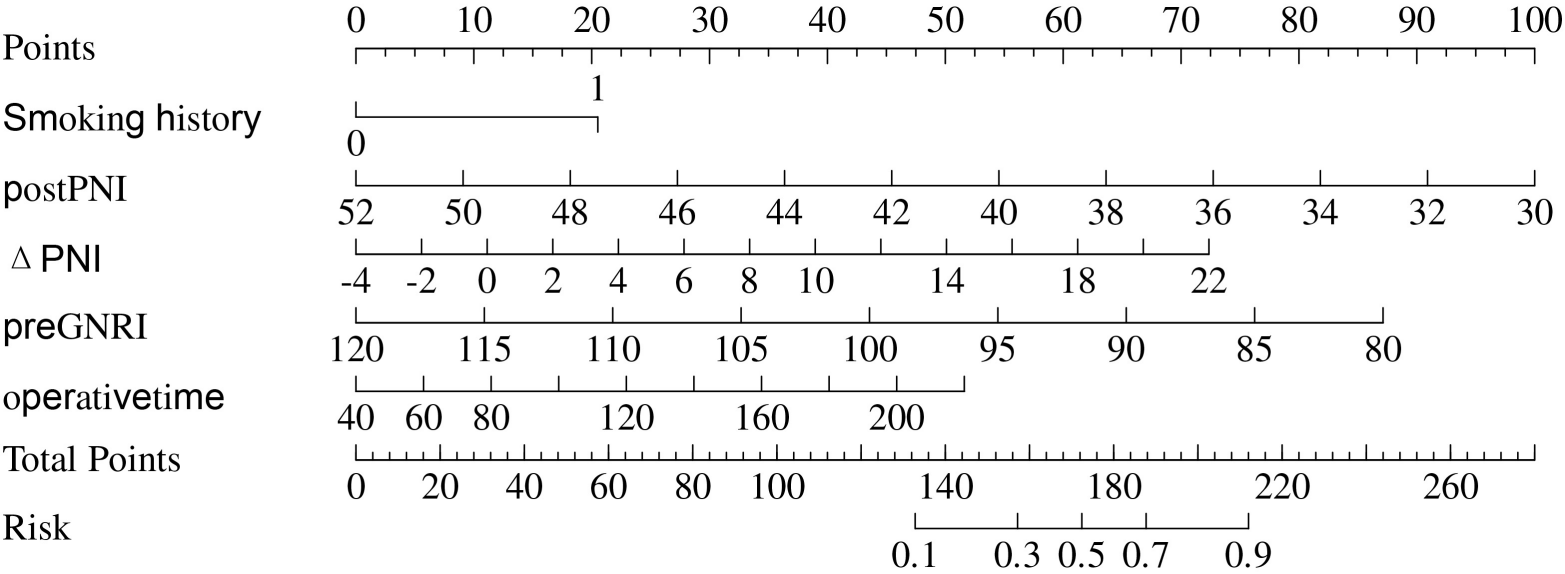
Flowchart of this study.

This retrospective study involved elderly patients who underwent pneumonectomy and were diagnosed with primary non-small cell lung cancer (NSCLC) through postoperative pathology at our hospital between January 2022 and December 2025. All patients received standardized treatment according to the Clinical Practice Guidelines for Thoracic Surgery for Lung Cancer, which were developed by the Chinese Thoracic Surgery Association. Our hospital's experienced surgical team performed the surgeries using standard surgical techniques. The inclusion criteria are as follows: (1) age ≥65 years, (2) underwent anatomical lung resection (including lobectomy, segmentectomy, wedge resection, or pneumonectomy), (3) aimed for radical (R0) resection, (4) diagnosed with primary NSCLC through postoperative pathology, and (5) had complete clinical pathology and follow-up data. Exclusion criteria: (1) Received neoadjuvant chemoradiotherapy, targeted therapy, or immunotherapy before surgery; (2) Had other malignancies or a history of malignancies; (3) Had severe infections, hematological disorders, autoimmune diseases, or long-term use of glucocorticoids; (4) Had severe preoperative liver or kidney dysfunction; (5) Underwent emergency surgery or palliative surgery.Patients with any missing clinical or follow-up data were excluded (*n* = 102, see Figure 1). Thus, no missing data imputation was required. This study included a total of 364 eligible patients who were randomly divided into training and validation sets at a ratio of 7:3 (*n* = 254 and *n* = 110, respectively).

### Study protocol

This study was approved by the hospital ethics committee (2026-03-wen-01) and adhered to the principles of the Declaration of Helsinki (revised in 2013) (17). Informed consent from patients was waived, and the study was conducted and reported in accordance with the Standards for Reporting of Case-Control Studies (STROCSS). Sample size estimation for this study was based on the primary research objective of constructing a predictive model for postoperative cardiopulmonary complications. According to the empirical rule (EPV ≥ 10) and literature reports, the estimated incidence of postoperative cardiopulmonary complications in elderly lung cancer patients is 25% to 30%. The plan is to include five to eight predictive variables, which requires at least 50 to 80 patients with complications. Considering a data missing rate of 10% to 15%, PASS 15.0 software estimated the minimum sample size to be 320 cases (test power 0.80, α = 0.05). Ultimately, this study included 364 cases, 97 of which (26.65%) experienced complications. The actual EPV was approximately 19.4:1, meeting the modeling requirements. The research flowchart is shown in Figure 1.

### Definition of postoperative cardiopulmonary complications

This study used the diagnostic criteria for cardiovascular and pulmonary complications described in the published literature, the STS/ESTS (18) criteria, and recorded postoperative cardiovascular and pulmonary complications (persistent lung leak, pulmonary infection, arrhythmia, acute myocardial infarction, pulmonary embolism, heart failure, empyema, bronchopleural fistula, secondary endotracheal intubation, death, etc.). The main outcome measure is the occurrence of complications within 30 days after lung resection.

### Data collection and potential predictive factors

Based on relevant literature and clinical experience, this study collected clinical data retrospectively from eligible patients through the hospital's electronic medical record system, quality control registration system, and laboratory testing system. The collected data included the following: age; gender; smoking and drinking history; BMI; underlying diseases (e.g., COPD, hypertension, diabetes, hyperlipidemia, and cerebral infarction); surgical procedure (e.g., lobectomy or partial lung resection); operation time; and laboratory indicators (e.g., blood routine, liver and kidney function, coagulation function, and blood lipids) 1 week before and 1 day after surgery. Using the blood routine results, we calculated the preoperative and postoperative prognostic nutritional index (PNI) and geriatric nutritional risk index (GNRI) using the following formulas: PNI = 10 × serum albumin (g/dL) + 0.005 × peripheral blood lymphocyte count (mm3) and GNRI = 1.489 × serum albumin (g/L) + 41.7 × (actual weight/ideal weight). ΔPNI was calculated as preoperative PNI minus postoperative PNI (i.e., ΔPNI = prePNI – postPNI). Thus, a positive ΔPNI indicates a postoperative decline in nutritional-immune status, and a larger ΔPNI represents a greater decline.

### Development and evaluation of column chart models

A total of 364 patients were included in this study and divided at a 7:3 ratio into a training set (*n* = 254) and a validation set (*n* = 110). Candidate predictors potentially associated with postoperative cardiopulmonary complications were selected based on clinical relevance and prior literature. Univariate logistic regression was first performed using the training set data, and variables with a *P* value < 0.05 were entered into the multivariable analysis. A backward stepwise selection procedure using the likelihood ratio test was then applied, with a retention criterion of *P* < 0.05. Multicollinearity among the selected variables was assessed using the variance inflation factor (VIF), with a VIF < 5 indicating no significant collinearity. The final model was constructed using the identified independent predictors. Model goodness-of-fit was evaluated using the Hosmer-Lemeshow test to assess consistency between predicted probabilities and observed outcomes. Discriminatory ability and calibration were assessed using receiver operating characteristic (ROC) curves, area under the curve (AUC), concordance index (C-index), and calibration plots. Clinical net benefit was evaluated using decision curve analysis (DCA). Discriminatory performance was further assessed via 10-fold cross-validation, and calibration was validated using 1,000 bootstrap resamples.

### Statistical analysis

All statistical analyses were performed using R software (version 4.4.0) with the rms, pROC, ggplot2, and DCA packages. Continuous variables with a normal distribution were expressed as mean ± standard deviation, and those with a skewed distribution were described as median (interquartile range); categorical variables were presented as counts (percentages). Candidate predictors were selected based on clinical relevance and prior literature. Univariate logistic regression was performed using the training set, and variables with *P* < 0.05 were entered into multivariable analysis; a backward stepwise selection procedure using the likelihood ratio test was applied with a retention criterion of *P* < 0.05. Multicollinearity was assessed using the variance inflation factor (VIF), with VIF < 5 indicating no significant collinearity. The predictive nomogram was constructed using the rms package based on the independent predictors identified in the final model. Model performance was evaluated using the concordance index (C-index), area under the curve (AUC), calibration curves, and decision curve analysis (DCA), and the Hosmer-Lemeshow test was used to assess calibration. Internal validation was performed using 10-fold cross-validation for discrimination and 1,000 bootstrap resamples for calibration. All statistical tests were two-tailed, and P < 0.05 was considered statistically significant.

## Results

### Patient characteristics

As shown in Table 1, 97 patients developed cardiopulmonary complications after surgery. Baseline characteristics were well balanced between the training and validation sets, with no significant differences (P > 0.05). Detailed patient baseline information is provided in Tables 1, 2.

**Table 1:** Patient demographics and baseline characteristics.

Characteristic	Complication		*p*
	No (*n* = 267)	Yes (*n* = 97)	
Sex (male)	111 (41.6%)	41 (42.3%)	0.905
Smoking history (yes)	65 (24.3%)	41 (42.3%)	< 0.001
Drinking history (yes)	42 (15.7%)	15 (15.5%)	0.951
ASA (3–4 points)	47 (17.6%)	21 (21.6%)	0.381
Diabetes history (yes)	22 (8.2%)	7 (7.2%)	0.750
History of hypertension (yes)	37 (13.9%)	21 (21.6%)	0.073
Hyperlipidemia (yes)	22 (8.2%)	11 (11.3%)	0.362
History of coronary heart disease (yes)	30 (11.2%)	9 (9.3%)	0.593
COPD (Yes)	29 (10.9%)	12 (12.4%)	0.687
Lobectomy (Yes)	89 (33.3%)	50 (51.5%)	0.002
Stages (Phase III-IV)	7 (2.6%)	3 (3.1%)	0.730
Age	68.0 (64.0, 71.0)	68.0 (64.0, 71.0)	0.521
BMI	23.4 (21.3, 25.6)	24.3 (21.9, 26.2)	0.073
preWBC	5.31 (4.29, 6.43)	5.51 (4.84, 6.71)	0.067
preRBC	4.41 (4.10, 4.75)	4.36 (4.10, 4.72)	0.412
preHB	133 ± 15	130 ± 17	0.124
preP	170 (137, 210)	179 (144, 209)	0.342
preN	3.16 (2.54, 4.16)	3.49 (2.90, 4.36)	0.016
preL	1.50 (1.18, 1.79)	1.44 (1.11, 1.85)	0.693
preM	0.32 (0.25, 0.41)	0.34 (0.28, 0.40)	0.179
preCRP	1.4 (0.8, 2.7)	1.8 (0.9, 4.0)	0.113
preDD	0.25 (0.17, 0.42)	0.30 (0.21, 0.40)	0.185
preALB	41.1 ± 3.1	39.5 ± 3.2	< 0.001
postALB	36.08 ± 2.95	35.41 ± 2.53	0.036
prePNI	49.2 ± 4.3	49.5 ± 4.8	0.687
postPNI	42.2 (39.8, 44.4)	39.6 (37.3, 41.0)	< 0.001
ΔPNI	7.0 ± 3.9	10.2 ± 4.4	< 0.001
preGNRI	101.9 ± 5.1	99.6 ± 5.3	< 0.001
postGNRI	94.4 ± 4.8	93.5 ± 4.4	0.114
ΔGNRI	7.5 ± 5.1	6.1 ± 5.0	0.019
eGFR	100 (91, 110)	98 (89, 108)	0.281
preCr	61 (52, 72)	63 (54, 71)	0.707
preUr	5.30 (4.40, 6.40)	5.30 (4.30, 6.30)	0.987
preUA	320 (270, 368)	324 (267, 372)	0.944
Triglyceride	0.85 (0.70, 1.19)	0.85 (0.72, 1.31)	0.905
Cholesterol	4.45 (3.82, 4.80)	4.54 (3.96, 4.99)	0.258
HDL	1.34 (1.18, 1.59)	1.35 (1.15, 1.66)	0.884
LDL	2.72 ± 0.59	2.83 ± 0.61	0.152
Operativetime	103 (72, 127)	113 (79, 133)	0.074

BMI, (Body Mass Index, a measure of body fat based on weight and height); WBC, (White Blood Cell Count, the number of white blood cells in the blood); RBC, (Red Blood Cell Count, the number of red blood cells); HB, (Hemoglobin Concentration, the level of hemoglobin in the blood); P, (Platelet Count, the number of platelets); N, (Neutrophil Percentage, the proportion of neutrophils among white blood cells); L, (Lymphocyte Percentage, the proportion of lymphocytes); M, (Monocyte Percentage, the proportion of monocytes); PNI, prognostic nutritional index; GNRI, geriatric nutritional risk index; CRP, (C-reactive Protein Concentration, an acute phase protein indicating inflammation); DD, (D-dimer Concentration, a fibrin degradation product suggesting potential thrombosis); ALB, (Albumin Concentration, a major plasma protein, with low levels indicating malnutrition or inflammation); eGFR, (Estimated Glomerular Filtration Rate, a measure of kidney function); Cr, (Creatinine Concentration, a key renal function indicator); Urea, (Urea Nitrogen Concentration, another important renal function indicator); UA, (Uric Acid Concentration, the final product of purine metabolism, with high levels indicating hyperuricemia), and Operative Time (the duration of the surgery). The terms pre and post refer to measurements taken before and after surgery, respectively, while Δ indicates the change from pre- to post-surgery.

**Table 2:** Comparison of training and validation sets.

Characteristic	Total (*n* = 364)	Test (*n* = 110)	Train (*n* = 254)	*P*
Age	67.60 ± 4.73	68.32 ± 4.69	67.29 ± 4.73	0.056
BMI	23.75 ± 3.16	23.40 ± 2.82	23.89 ± 3.29	0.174
preWBC	5.65 ± 1.80	5.68 ± 1.75	5.64 ± 1.82	0.868
preRBC	4.44 ± 0.53	4.48 ± 0.58	4.42 ± 0.51	0.336
preHB	132.58 ± 15.37	133.99 ± 14.04	131.96 ± 15.90	0.249
preP	176.96 ± 54.88	175.56 ± 50.70	177.57 ± 56.67	0.749
preN	3.59 ± 1.59	3.64 ± 1.56	3.57 ± 1.61	0.693
preL	1.57 ± 0.75	1.53 ± 0.55	1.59 ± 0.82	0.445
preM	0.35 ± 0.13	0.35 ± 0.13	0.35 ± 0.13	0.932
preCRP	4.73 ± 17.81	5.75 ± 23.36	4.29 ± 14.81	0.472
preDD	0.37 ± 0.42	0.37 ± 0.41	0.36 ± 0.42	0.866
preALB	40.67 ± 3.22	40.68 ± 3.13	40.67 ± 3.26	0.970
postALB	35.90 ± 2.86	35.83 ± 2.86	35.93 ± 2.87	0.772
prePNI	49.31 ± 4.40	49.02 ± 4.51	49.43 ± 4.35	0.412
postPNI	41.47 ± 3.65	41.46 ± 4.03	41.48 ± 3.48	0.979
ΔPNI	7.83 ± 4.28	7.55 ± 4.27	7.95 ± 4.28	0.412
preGNRI	101.26 ± 5.23	101.25 ± 5.04	101.26 ± 5.32	0.988
postGNRI	94.15 ± 4.73	94.03 ± 4.50	94.20 ± 4.83	0.752
ΔGNRI	7.11 ± 5.12	7.22 ± 5.47	7.06 ± 4.97	0.783
eGFR	98.91 ± 15.43	99.88 ± 15.54	98.49 ± 15.40	0.429
preCr	63.74 ± 16.28	62.60 ± 17.11	64.24 ± 15.91	0.379
preUr	5.53 ± 1.65	5.29 ± 1.84	5.64 ± 1.55	0.065
preUA	325.74 ± 81.35	317.96 ± 87.16	329.10 ± 78.64	0.231
Triglyceride	1.10 ± 0.81	1.06 ± 0.61	1.12 ± 0.89	0.501
Cholesterol	4.41 ± 0.84	4.39 ± 0.87	4.41 ± 0.83	0.849
HDL	1.40 ± 0.31	1.39 ± 0.31	1.40 ± 0.31	0.614
LDL	2.75 ± 0.60	2.74 ± 0.64	2.76 ± 0.57	0.816
Operativetime	104.21 ± 36.56	100.19 ± 34.24	105.96 ± 37.45	0.167
Sex (male)	152 (41.76)	49 (44.55)	103 (40.55)	0.478
Smoking history (yes)	106 (29.12)	36 (32.73)	70 (27.56)	0.319
Drinking history (yes)	57 (15.66)	19 (17.27)	38 (14.96)	0.577
ASA (3–4 points)	68 (18.68)	25 (22.73)	43 (16.93)	0.192
Diabetes history (yes)	29 (7.97)	9 (8.18)	20 (7.87)	0.921
History of hypertension (yes)	58 (15.93)	17 (15.45)	41 (16.14)	0.869
Hyperlipidemia (yes)	33 (9.07)	8 (7.27)	25 (9.84)	0.433
History of coronary heart disease (yes)	39 (10.71)	13 (11.82)	26 (10.24)	0.654
COPD (yes)	41 (11.26)	14 (12.73)	27 (10.63)	0.561
Lobectomy (yes)	139 (38.19)	40 (36.36)	99 (38.98)	0.638
Stages (phase III-IV)	10 (2.75)	4 (3.64)	6 (2.36)	0.739
Complication (yes)	97 (26.65)	30 (27.27)	67 (26.38)	0.859

BMI, (Body Mass Index, a measure of body fat based on weight and height); WBC, (White Blood Cell Count, the number of white blood cells in the blood); RBC, (Red Blood Cell Count, the number of red blood cells); HB, (Hemoglobin Concentration, the level of hemoglobin in the blood); P, (Platelet Count, the number of platelets); N, (Neutrophil Percentage, the proportion of neutrophils among white blood cells); L, (Lymphocyte Percentage, the proportion of lymphocytes); M, (Monocyte Percentage, the proportion of monocytes); PNI, prognostic nutritional index; GNRI: geriatric nutritional risk index; CRP (C-reactive Protein Concentration, an acute phase protein indicating inflammation); DD, (D-dimer Concentration, a fibrin degradation product suggesting potential thrombosis); ALB, (Albumin Concentration, a major plasma protein, with low levels indicating malnutrition or inflammation); eGFR, (Estimated Glomerular Filtration Rate, a measure of kidney function), Cr (Creatinine Concentration, a key renal function indicator); Urea, (Urea Nitrogen Concentration, another important renal function indicator); UA, (Uric Acid Concentration, the final product of purine metabolism, with high levels indicating hyperuricemia), and Operative Time (the duration of the surgery). The terms pre and post refer to measurements taken before and after surgery, respectively, while Δ indicates the change from pre- to post-surgery.

### Screening predictive factors

Univariate analysis identified a total of nine candidate variables with P < 0.05: smoking history, hypertension history, BMI, lobectomy, preoperative albumin, postoperative PNI, ΔPNI, preoperative GNRI, and operative time. However, collinearity diagnosis revealed severe multicollinearity for BMI (VIF = 38.77), preoperative albumin (VIF = 12.66), preoperative GNRI (VIF = 14.09), and operative time (VIF = 37.68). After backward stepwise regression excluded the collinear variables (BMI, preoperative albumin, hypertension history, and lobectomy), the final model containing smoking history, preoperative GNRI, postoperative PNI, ΔPNI, and operative time was reassessed for multicollinearity. All variance inflation factors in this final model were below 2, indicating no significant collinearity.

Multivariate logistic regression analysis showed that all five of these variables were independent risk factors (*P* < 0.05): smoking history (OR = 3.12), preoperative GNRI (OR = 0.89), postoperative PNI (OR = 0.78), ΔPNI (OR = 1.17), and operative time (OR = 1.02). These variables can serve as core indicators for assessing the risk of postoperative cardiopulmonary complications in elderly patients with non-small cell lung cancer.For ΔPNI, the OR was 1.17 (95% CI: 1.07–1.28, *P* < 0.001), meaning that each 1-point increase in the postoperative decline of PNI (i.e., a larger ΔPNI) was associated with a 17% higher risk of cardiopulmonary complications (Table 3).

**Table 3:** Single factor and multiple factor logistic regression analyses used for screening predictive factors.

Characteristic	Univariate analysis		Multivariate analysis	
	*P*	OR (95% CI)	*P*	OR (95% CI)
Sex (male)	0.735	0.91 (0.51 ~ 1.60)		
Smoking history (yes)	< 0.001	2.72 (1.50 ~ 4.93)	0.002	3.12 (1.52 ~ 6.43)
Drinking history (yes)	0.431	1.35 (0.64 ~ 2.86)		
ASA (3–4 points)	0.803	1.10 (0.53 ~ 2.29)		
Diabetes history (yes)	0.102	0.29 (0.07 ~ 1.28)		
History of hypertension (yes)	0.048	2.03 (1.01 ~ 4.10)		
Hyperlipidemia (yes)	0.254	1.66 (0.70 ~ 3.96)		
History of coronary heart disease (yes)	0.386	0.64 (0.23 ~ 1.76)		
COPD (yes)	0.955	0.97 (0.39 ~ 2.42)		
Lobectomy (yes)	0.010	2.10 (1.19 ~ 3.70)		
Stages (Phase III-IV)	0.697	1.41 (0.25 ~ 7.87)		
Age	0.839	1.01 (0.95 ~ 1.07)		
BMI	0.048	1.09 (1.01 ~ 1.19)		
preWBC,	0.502	1.05 (0.91 ~ 1.22)		
preRBC	0.413	0.79 (0.46 ~ 1.38)		
preHB	0.173	0.99 (0.97 ~ 1.01)		
preP	0.261	1.00 (1.00 ~ 1.01)		
preN	0.339	1.08 (0.92 ~ 1.28)		
preL	0.383	1.15 (0.84 ~ 1.59)		
preM	0.532	1.94 (0.24 ~ 15.53)		
preCRP	0.686	1.00 (0.97 ~ 1.02)		
preDD	0.884	0.95 (0.48 ~ 1.88)		
preALB	0.003	0.87 (0.80 ~ 0.95)		
postALB	0.212	0.94 (0.85 ~ 1.04)		
prePNI	0.268	1.04 (0.97 ~ 1.11)		
postPNI	< 0.001	0.77 (0.70 ~ 0.85)	< 0.001	0.78 (0.69 ~ 0.88)
ΔPNI	< 0.001	1.22 (1.13 ~ 1.32)	< 0.001	1.17 (1.07 ~ 1.28)
preGNRI	0.021	0.94 (0.89 ~ 0.99)	< 0.001	0.89 (0.83 ~ 0.95)
postGNRI	0.531	0.98 (0.93 ~ 1.04)		
ΔGNRI	0.061	0.95 (0.89 ~ 1.00)		
eGFR	0.723	1.00 (0.98 ~ 1.01)		
preCr	0.733	1.00 (0.98 ~ 1.01)		
preUr	0.223	0.89 (0.74 ~ 1.07)		
preUA	0.462	1.00 (1.00 ~ 1.00)		
Triglyceride	0.680	0.93 (0.67 ~ 1.30)		
Cholesterol	0.303	1.19 (0.85 ~ 1.68)		
HDL	0.712	1.19 (0.48 ~ 2.94)		
LDL	0.214	1.36 (0.84 ~ 2.23)		
Operative time	0.029	1.01 (1.01 ~ 1.02)	0.002	1.02 (1.01 ~ 1.03)

OR, Odds Ratio; CI, Confidence Interval; BMI, (Body Mass Index, a measure of body fat based on weight and height); WBC, (White Blood Cell Count, the number of white blood cells in the blood); RBC, (Red Blood Cell Count, the number of red blood cells); HB, (Hemoglobin Concentration, the level of hemoglobin in the blood); P, (Platelet Count, the number of platelets); N, (Neutrophil Percentage, the proportion of neutrophils among white blood cells); L, (Lymphocyte Percentage, the proportion of lymphocytes); M, (Monocyte Percentage, the proportion of monocytes); PNI, prognostic nutritional index; GNRI: geriatric nutritional risk index; CRP, (C-reactive Protein Concentration, an acute phase protein indicating inflammation); DD, (D-dimer Concentration, a fibrin degradation product suggesting potential thrombosis); ALB, (Albumin Concentration, a major plasma protein, with low levels indicating malnutrition or inflammation); eGFR, (Estimated Glomerular Filtration Rate, a measure of kidney function); Cr, (Creatinine Concentration, a key renal function indicator); Urea, (Urea Nitrogen Concentration, another important renal function indicator); UA, (Uric Acid Concentration, the final product of purine metabolism, with high levels indicating hyperuricemia), and Operative Time (the duration of the surgery). The terms pre and post refer to measurements taken before and after surgery, respectively, while Δ indicates the change from pre- to post-surgery. Variables with *P* < 0.05 in univariate analysis were entered into multivariable backward stepwise regression. Variables with *P* < 0.05 were retained in the final model.

### Develop personalized prediction models

**Figure 2:**
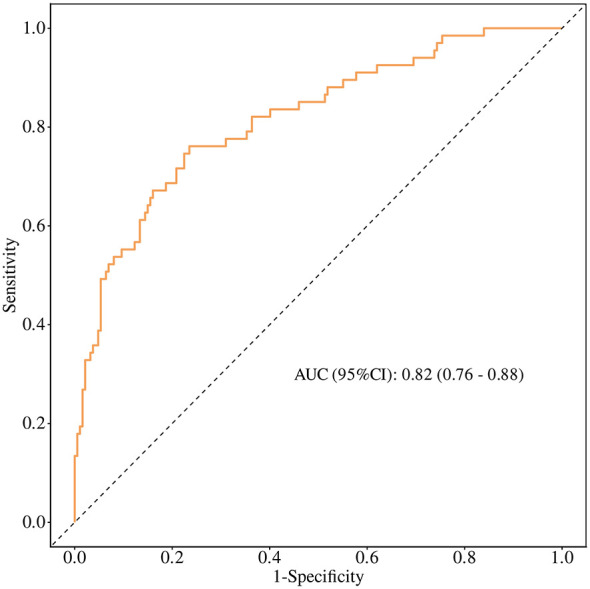
Column chart for research.

A nomogram model for predicting postoperative cardiopulmonary complications in elderly patients with non-small cell lung cancer was constructed using the rms package in R software. This model is based on five independent risk factors identified through multifactorial logistic regression analysis: smoking history, preoperative GNRI, postoperative PNI, ΔPNI, and operation duration (Figure 2). This nomogram quantifies the impact of each risk factor. Operation duration, ΔPNI, and preoperative GNRI have the highest scoring weights, followed by smoking history and postoperative PNI. When used, the specific values of each indicator for a patient are assigned corresponding scores on the appropriate scale. The total score is obtained by adding the individual scores and mapping them to the risk axis. This allows for an intuitive prediction of the probability of postoperative cardiopulmonary complications in individual patients. The nomogram visualizes the regression model, facilitating rapid and convenient risk assessment for clinicians.

### Prediction model validation and calibration

**Figure 3:**
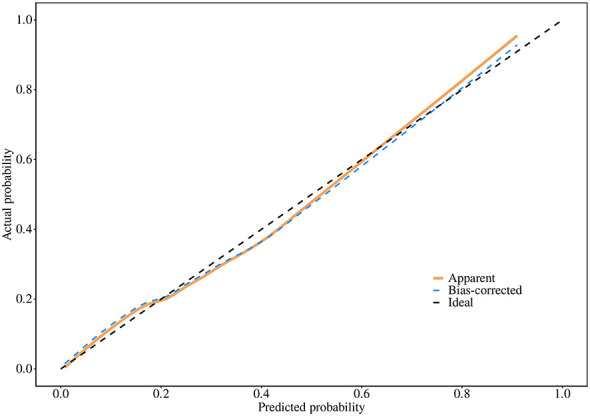
ROC curve of the training set.

**Figure 4:**
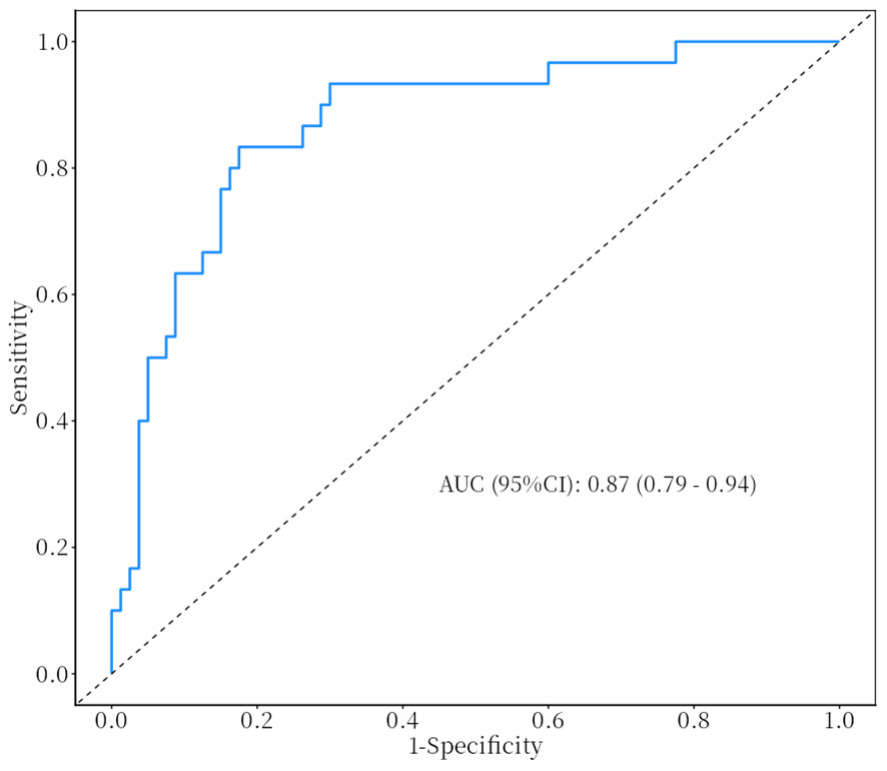
ROC curve of validation set.

We evaluated the discriminatory ability of the model using the receiver operating characteristic (ROC) curve. The results showed that the area under the curve (AUC) for the training set was 0.82 [95% confidence interval (CI): 0.76–0.88] and for the validation set was 0.87 (95% CI: 0.79–0.94). These results indicate that the model exhibited good predictive ability and stable performance in both independent datasets (see Figures 3, 4). The detailed diagnostic performance of the nomogram is summarized in Table 4.

**Table 4:** Diagnostic performance of the model in training and test sets.

Data	AUC (95% CI)	Accuracy (95% CI)	Sensitivity (95% CI)	Specificity (95% CI)	PPV (95% CI)	NPV (95% CI)	Cut off
Train	0.82 (0.76–0.88)	0.76 (0.71–0.81)	0.76 (0.66–0.86)	0.76 (0.70–0.83)	0.54 (0.44–0.64)	0.90 (0.85–0.95)	0.288
Test	0.87 (0.79–0.94)	0.80 (0.71–0.87)	0.83 (0.70–0.97)	0.79 (0.70–0.88)	0.60 (0.45–0.74)	0.93 (0.86–0.99)	0.288

**Figure 5:**
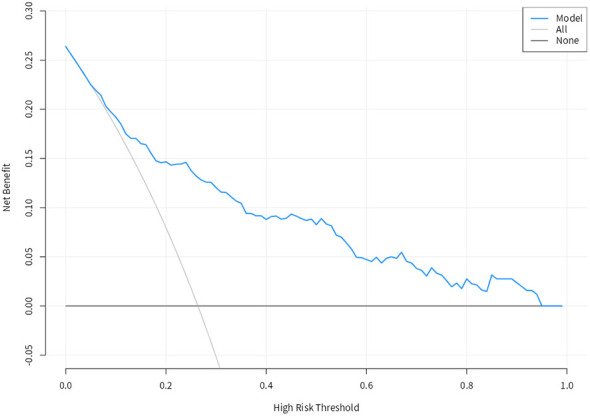
DCA curve in training cohort.

**Figure 6:**
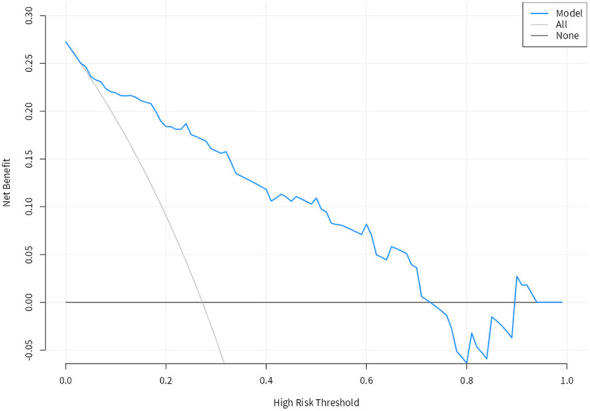
DCA curve in internal test cohort.

Decision curve analysis revealed that, across a broad spectrum of risk thresholds, the clinical net benefit of the nomogram model surpasses the “all intervention” and “no intervention” alternatives, suggesting its substantial clinical application value (see Figures 5, 6).

**Figure 7:**
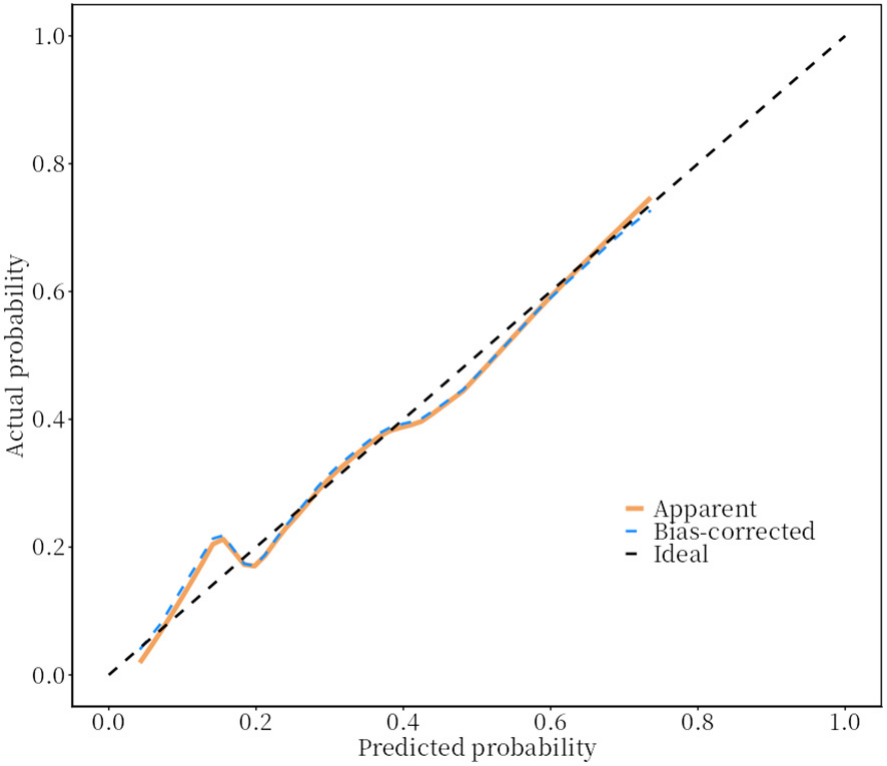
Calibration curve in training cohort.

**Figure 8:**
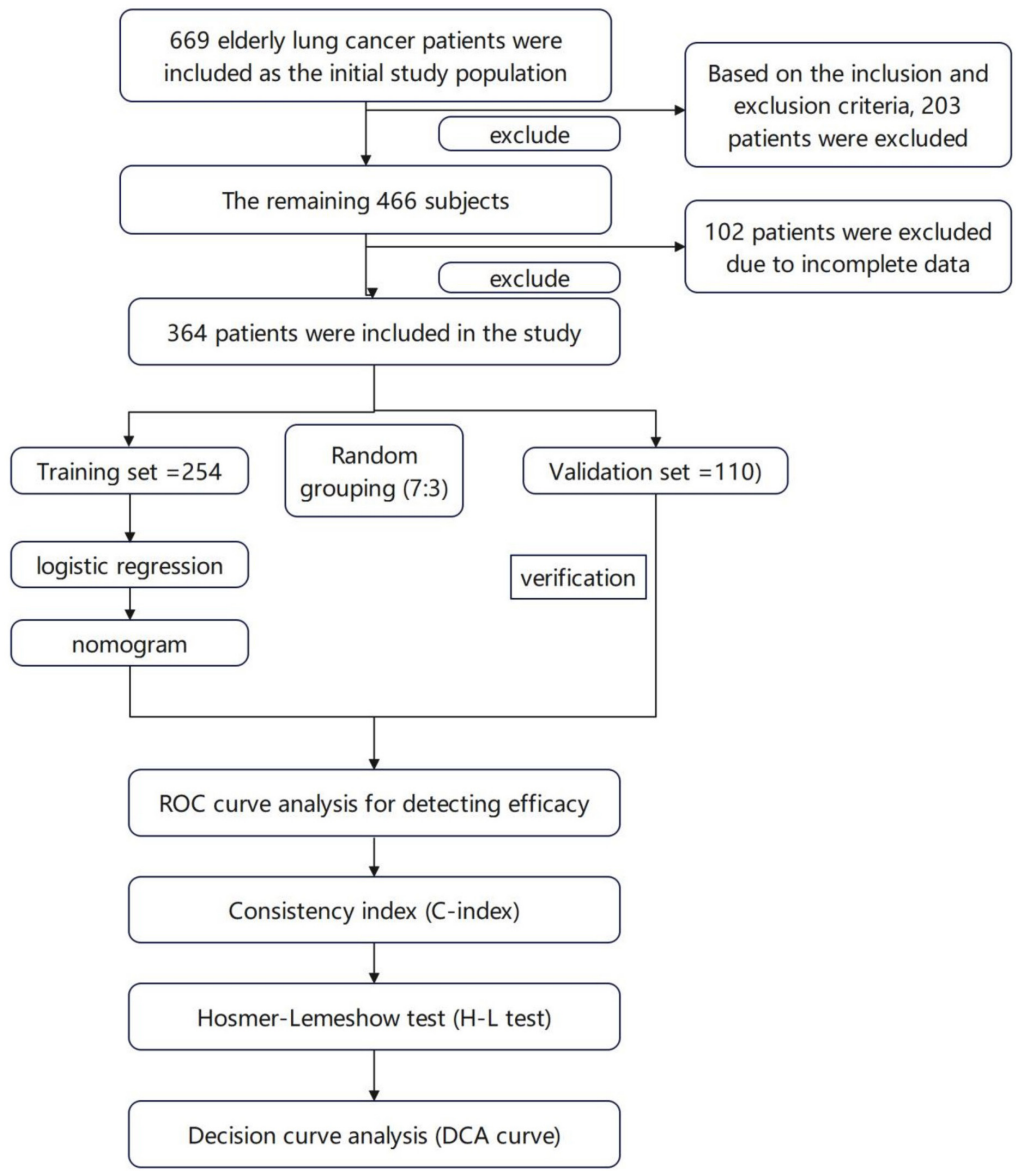
Calibration curve in internal test cohort.

Calibration was evaluated using the Hosmer-Lemeshow goodness-of-fit test. For the training set, the Hosmer-Lemeshow test yielded χ^2^ = 6.102, df = 8, and *P* = 0.636. For the validation set, the test yielded χ^2^ = 2.017, df = 8, and *P* = 0.981. These results suggest that there is no significant difference between the model's predicted and observed probabilities, indicating good model fit. The calibration curve visually demonstrates this consistency (see Figures 7, 8). After 1,000 rounds of bootstrap resampling internal validation, the calibrated calibration curve closely matched the ideal curve, suggesting reliable calibration of the model.

## Discussion

This study successfully developed and validated a nomogram specifically designed to predict the risk of cardiopulmonary complications in elderly patients with non-small cell lung cancer undergoing pneumonectomy. The model incorporates five independent risk factors: smoking history, preoperative geriatric nutritional risk index, postoperative prognostic nutritional index, perioperative change in prognostic nutritional index, and operation duration. The model exhibits superior discriminatory power and good calibration in both the internal training and validation sets. The core value of this tool lies in its ability to provide personalized and visual information. Transforming a complex regression model into an intuitive scoring chart enables clinicians to conveniently and quickly quantify the perioperative risks of this high-risk elderly population. In the context of the increasingly popular concept of accelerated rehabilitation surgery, such predictive tools are expected to provide evidence-based support for precise preoperative assessment, intraoperative optimization management, and postoperative focused monitoring. This will promote the advancement of geriatric lung cancer surgery toward a more precise, safer, and individualized medical model.

The predictive factors identified by the model have clear biological and clinical significance. Assessing nutritional and inflammatory status is central. Both a reduction in preoperative GNRI and a reduction in postoperative PNI are independently associated with an increased risk of complications, which highlights the importance of nutritional reserves and immune capacity in tolerating surgical trauma and promoting postoperative recovery. Serum albumin, a key component of GNRI and PNI, is a marker of nutritional status and a negative indicator of systemic inflammation. Multiple studies have confirmed that hypoalbuminemia is associated with a poor prognosis in patients with various cancers, including non-small cell lung cancer (NSCLC) (19). Similarly, composite indicators composed of albumin and lymphocyte counts, such as the hemoglobin-albumin-lymphocyte-platelet score, have been shown to be associated with survival and pathological characteristics in patients with NSCLC (20). This study expands the use of inflammatory-nutritional composite indicators to predicting perioperative complications. This finding reinforces the idea that the inflammatory burden index is a superior systemic inflammatory biomarker that can predict NSCLC prognosis (21). This further emphasizes the important roles of systemic inflammation and malnutrition in managing lung cancer patients comprehensively.

This study also confirmed the importance of two more traditional perioperative risk factors: smoking history and duration of surgery. Smoking is a major risk factor for comorbidities, such as chronic obstructive pulmonary disease (COPD), which directly affects pulmonary reserve and increases the risk of postoperative complications, such as pulmonary infection and respiratory failure. A prolonged surgery is often associated with increased surgical complexity, aggravated tissue trauma, and prolonged anesthesia exposure. These factors may exacerbate the systemic inflammatory response and physiological stress. A study on accelerated rehabilitation surgical pathways showed that optimizing perioperative processes can shorten hospital stays and reduce cardiopulmonary complication rates in patients undergoing thoracoscopic lobectomy (22), indirectly supporting the importance of controlling surgery-related factors. Meanwhile, research on the timing of surgery suggests that, for clinical stage I non-small cell lung cancer, delaying surgery by more than 12 weeks may be associated with an increased risk of recurrence and poorer survival outcomes (23). While this study focuses on complications rather than oncological outcomes, both emphasize the importance of performing efficient and precise surgical interventions when medical conditions permit.

One of the most distinctive findings of this study is that the dynamic changes in perioperative PNI (ΔPNI) have been established as an independent predictor. Unlike a single preoperative or postoperative static value, ΔPNI captures nutritional-inflammatory fluctuations during a patient's early recovery process after undergoing surgical trauma from their preoperative baseline state. A negative increase in PNI indicates significant short-term deterioration in nutritional and immune status compared to preoperative conditions, which may signal severe surgical stress response, insufficient tissue repair capacity, or potential infection. Focusing on these dynamic changes provides a new perspective for evaluating patients' tolerance to surgery and recovery potential. Similarly, in the systemic treatment of advanced non-small cell lung cancer, dynamic changes in albumin concentration during treatment have been found to indicate disease activity or treatment response (1). Therefore, monitoring the dynamic trajectory of key biomarkers during the perioperative period may more accurately reflect patients' physiological status and prognosis than absolute values at a single time point.

The nomogram model, which integrates the aforementioned factors, shows promise for clinical application. Balancing surgical benefits and perioperative risks remains a significant challenge in surgical decision-making for elderly patients with non-small cell lung cancer. Evidence suggests that elderly patients undergoing anatomical lung resection at experienced centers do not experience significantly compromised short-term outcomes (24). However, identifying higher-risk subgroups is crucial for optimizing resource allocation. This model provides a quantitative tool that helps surgeons communicate risks more effectively with patients and their families before surgery and offers a basis for developing individualized perioperative management plans. For example, patients predicted to be at high risk by the model could receive enhanced preoperative rehabilitation, optimized anesthesia plans, and more minimally invasive surgical techniques. They could also be admitted to a higher-level intensive care unit after surgery. This risk-based, stratified management strategy is similar to the approach of using “textbook outcomes” as a composite indicator for comprehensive performance evaluation in thoracic surgery (25), as both aim to enhance overall diagnostic and treatment quality by integrating multidimensional indicators.

## Limitations

Limitations of this Study: First, as a single-center retrospective study, the conclusions are inevitably influenced by selection bias and confounding factors. Despite the use of strict inclusion and exclusion criteria and multivariable adjustments, some unmeasured confounding factors may still exist. Second, the relatively limited sample size from a single institution limits the generalizability of the model. Although internal validation shows good performance, the model's external validity in different geographical regions and institutions with varying levels of medical care has not been verified. Third, the predictive factors relied on by the model, such as PNI and GNRI, depend on laboratory tests performed at specific time points, and these tests may be affected by the methods used and by short-term fluctuations in individuals. Future prospective, multicenter, large-sample studies are needed to validate and optimize this predictive model further.

## Conclusion

This study successfully developed a nomogram model to predict the risk of postoperative cardiopulmonary complications in elderly patients with non-small cell lung cancer (NSCLC). The model incorporates five independent risk factors, such as smoking history and preoperative and postoperative nutritional index (PNI), and has demonstrated good discrimination and calibration through internal validation. The model can serve as a visual tool to help clinicians quickly quantify and assess perioperative risks. The study confirms that nutritional status and surgery-related factors jointly influence postoperative recovery. Specifically, dynamic perioperative changes in PNI (ΔPNI) compensate for the limitations of traditional static indicators and provide a new perspective for risk assessment. This model aligns with the concept of accelerated rehabilitation surgery and can provide evidence-based support for preventing and controlling postoperative complications in elderly NSCLC patients.

## Data Availability

The original contributions presented in the study are included in the article/Supplementary material, further inquiries can be directed to the corresponding authors.

## References

[1] Sung H Ferlay J Siegel RL Laversanne M Soerjomataram I Jemal A . Global cancer statistics 2020: GLOBOCAN estimates of incidence and mortality worldwide for 36 cancers in 185 countries. CA Cancer J Clin. (2021) 71:209–49. doi: 10.3322/caac.21660 33538338

[2] Tang FH Wong HYT Tsang PSW Yau M Tam SY Law L . Recent advancements in lung cancer research: a narrative review. Transl Lung Cancer Res. (2025) 14:975–90. doi: 10.21037/tlcr-24-979 40248731 PMC12000946

[3] Sharma B Ganti AK. Promising trends in lung cancer care, but are we overlooking the majority. Aging. (2023) 15:8531–2. doi: 10.18632/aging.204662 37665677 PMC10522388

[4] Saftic I Bille A Asemota N Berjon dlVL Routledge T King J . Risks and rewards of the surgical treatment of lung cancer in octogenarians. Interact Cardiovasc Thorac Surg. (2021) 33:905–12. doi: 10.1093/icvts/ivab194 34436584 PMC8632787

[5] Kaprin A Pikin O Ryabov A Aleksandrov O Larionov D Garifullin A. Surgical intervention for lung cancer in patients aged 75 and above:potential associations with increased mortality rates-a single-center observational study. J Cardiothorac Surg. (2024) 19:471. doi: 10.1186/s13019-024-02922-5 39069611 PMC11285345

[6] Huang Q Rauniyar R Yang J Zhou C Cai D Chen-Yoshikawa TF . Risk stratification of postoperative pulmonary complications in elderly patients undergoing lung cancer resection:a propensity score-matched study. J Thorac Dis. (2023) 15:3908–18. doi: 10.21037/jtd-23-923 37559604 PMC10407502

[7] Okada S Shimomura M Ishihara S Ikebe S Furuya T Inoue M. Clinical significance of postoperative pulmonary complications in elderly patients with lung cancer. Interact Cardiovasc Thorac Surg. (2022) 35:ivac153. doi: 10.1093/icvts/ivac153 35640579 PMC9297523

[8] Lee YH Kim DH Kim J Lee J. Risk Assessment of Postoperative Pneumonia in Cancer Patients Using a Common Data Model. Cancers. (2022) 14:5988. doi: 10.3390/cancers14235988 36497470 PMC9740852

[9] Motono N Mizoguchi T Ishikawa M Iwai S Iijima Y Uramoto H. Analysis of risk factors of postoperative complication for non-small cell lung cancer. BMC Pulm Med. (2024) 24:333. doi: 10.1186/s12890-024-03054-1 38987733 PMC11238410

[10] Saetang M Kunapaisal T Wasinwong W Boonthum P Sriyanaluk B Nuanjun K. Predictors associated with Clavien-Dindo complications in lung cancer surgery:A retrospective cohort study. PLoS ONE. (2024) 19:e0316214. doi: 10.1371/journal.pone.0316214 39739759 PMC11687775

[11] Zhang Q Zhang L Jin Q He Y Wu M Peng H . The prognostic value of the GNRI in patients with stomach cancer undergoing surgery. J Pers Med. (2023) 13:155. doi: 10.3390/jpm13010155 36675816 PMC9861269

[12] Nakamura Y Nishimura T Kanemitsu E Nagata H Komori J Takada Y. Usefulness of the geriatric nutritional risk index (GNRI) as a predictor of postoperative complications after colorectal cancer surgery. Cureus. (2025) 17:e86268. doi: 10.7759/cureus.86268 40688986 PMC12275500

[13] Senger AS Dincer M Uzun O Gulmez S Avan D Ofluoglu CB . Impact of preoperative prognostic nutritional index levels on morbidity in colorectal cancer surgery. Ann Ital Chir. (2022) 92:422–6. 35190499

[14] Kato A Aoyama T Maezawa Y Hashimoto I Hara K Kazama K . Geriatric nutritional risk index is an independent prognostic factor for patients with esophageal cancer who receive curative treatment. Anticancer Res. (2024) 44:331–7. doi: 10.21873/anticanres.16816 38159974

[15] Takahashi M Aoyama A Hamaji M Sozu T Kobayashi M Nakagawa T . Clinical significance of the preoperative prognostic nutritional index in patients with resectable non-small cell lung cancer: a multicenter study. Surg Today. (2025) 55:918–29. doi: 10.1007/s00595-024-02987-8 39815110

[16] Hazer S Gülhan SSE Solak N Yenibertiz D Akilli MS Sayilir GE . The effect of prognostic nutritional index in postoperative infection following lobectomy in non-small cell lung cancer patients. Cureus. (2023) 15:e37611. doi: 10.7759/cureus.37611 37197130 PMC10184591

[17] World Medical Association General Assembly. World Medical Association Declaration of Helsinki: ethical principles for medical research involving human subjects. J Int Bioethique. (2004) 15:124–9. doi: 10.3917/jib.151.0124 15835069

[18] He Z Liu K Wu L Wei Q. Nomogram for predicting postoperative cardiopulmonary complications in non-small cell lung cancer based on systemic inflammatory markers: a retrospective study. J Inflamm Res. (2025) 18:8961–76. doi: 10.2147/JIR.S519449 40661188 PMC12258541

[19] Stares M Swan A Cumming K Ding T Leach J Stratton C . Hypoalbuminaemia as a prognostic biomarker of first-line treatment resistance in metastatic non-small cell lung cancer. Front Nutr. (2021) 8:734735. doi: 10.3389/fnut.2021.734735 34660664 PMC8517082

[20] Zhai B Chen J Wu J Yang L Guo X Shao J . Predictive value of the hemoglobin, albumin, lymphocyte, and platelet (HALP) score and lymphocyte-to-monocyte ratio (LMR) in patients with non-small cell lung cancer after radical lung cancer surgery. Ann Transl Med. (2021) 9:976. doi: 10.21037/atm-21-2120 34277776 PMC8267290

[21] Xie H Ruan G Wei L Deng L Zhang Q Ge Y . The inflammatory burden index is a superior systemic inflammation biomarker for the prognosis of non-small cell lung cancer. J Cachexia Sarcopenia Muscle. (2023) 14:869–78. doi: 10.1002/jcsm.13199 36852672 PMC10067487

[22] Forster C Doucet V Perentes JY Abdelnour-Berchtold E Zellweger M Faouzi M . Impact of an enhanced recovery after surgery pathway on thoracoscopic lobectomy outcomes in non-small cell lung cancer patients:a propensity score-matched study. Transl Lung Cancer Res. (2021) 10:93–103. doi: 10.21037/tlcr-20-891 33569296 PMC7867780

[23] Heiden BT Eaton DB Engelhardt KE Chang S Yan Y Patel MR . Analysis of delayed surgical treatment and oncologic outcomes in clinical stage i non-small cell lung cancer. JAMA Netw Open. (2021) 4:e2111613. doi: 10.1001/jamanetworkopen.2021.11613 34042991 PMC8160592

[24] Patella M Tessitore A Cianfarani A Minerva EM Cafarotti S. Early outcome of anatomical lung resection for non-small cell lung cancer in the elderly. Eur Rev Med Pharmacol Sci. (2021) 25:5129–36. doi: 10.26355/eurrev_202108_26525 34486687

[25] Kulshrestha S Vigneswaran WT Pawlik TM Baker MS Luchette FA Raad W . Assessment of textbook outcome after surgery for stage i/ii non-small cell lung cancer. Semin Thorac Cardiovasc Surg. (2022) 34:1351–9. doi: 10.1053/j.semtcvs.2021.08.009 34411699 PMC8848000

